# COVID-19: Identifying countries with indicators of success in responding to the outbreak

**DOI:** 10.12688/gatesopenres.13140.1

**Published:** 2020-06-18

**Authors:** David S. Kennedy, VK Vu, Hannah Ritchie, Rebecca Bartlein, Oliver Rothschild, Daniel G. Bausch, Max Roser, Anna C. Seale

**Affiliations:** 1 UK-Public Health Rapid Support Team, London School of Hygiene & Tropical Medicine/ Public Health England, London, UK; 2 Department of Epidemiology and Population Health, London School of Hygiene & Tropical Medicine, London, UK; 3 The Bill & Melinda Gates Foundation, Seattle, USA; 4 Our World in Data, Oxford, UK; 5 Oxford Martin School, University of Oxford, Oxford, UK; 6 Gates Ventures, Seattle, USA; 7 Department of Infectious and Tropical Diseases, London School of Hygiene & Tropical Medicine, London, UK

**Keywords:** COVID-19, pandemic, lessons, exemplars, outbreak, response, learning

## Abstract

**Background:** In designing responses to the COVID-19 pandemic, it is critical to understand what has already worked well. We aimed to identify countries with emerging success stories from whom policymakers might draw important lessons.

**Methods:** We developed a process to first include countries with large enough populations that results were unlikely to be due to chance, that had sufficient cases for response mechanisms to be tested, and that shared the necessary publicly available data. Within these countries, we looked at indicators suggesting success in terms of detecting disease, containing the outbreak, and treating those who were unwell. To support comparability, we measured indicators per capita (per million) and across time. We then used the indicators to identify three countries with emerging success stories to include some diversity in global region, population demographics and form of government.

**Results:** We identified 66 countries that met our inclusion criteria on 18 ^th^ May 2020. Several of these countries had indicators of success against the set indicators at different times in the outbreak. Vietnam had high levels of testing and successful containment with no deaths reported. South Korea had high levels of testing early in the outbreak, supporting containment. Germany had high levels of sustained testing and slower increases in cases and deaths than seen in other comparable settings.

**Conclusions:** At the time of our assessment, Vietnam and South Korea were able to contain the outbreak of COVID-19 and avoid the exponential growth in cases seen elsewhere. Germany had more cases and deaths, but was nevertheless able to contain and mitigate the outbreak. Despite the many limitations to the data currently available, looking at comparative data can help identify countries from whom we can draw lessons, so that countries can inform and adapt their strategies for success in response to COVID-19.

## Introduction

A cluster of cases of pneumonia of unknown aetiology were identified in December 2019 in Wuhan, China ^1^. These cases were found to be caused by a novel coronavirus, SARS-CoV-2. The outbreak of coronavirus disease 2019 (COVID-19) continued, and with cases identified across the world, the outbreak was declared a Public Health Emergency of International Concern by the World Health Organization (WHO) on January 30th, 2020 ^2^. At that time, there were 7,711 confirmed cases and 170 deaths in China, then the epicentre. Outside of China, 83 cases had been reported in 18 countries, with community transmission reported in three of these countries ^3^. Further spread led to the declaration of a pandemic on March 11th, 2020. By the end of April 2020, there were over 3 million cases and over 200,000 deaths reported in 213 countries, areas or territories ^2^.

In mid-March the WHO Strategic and Technical Advisory Group for Infectious Hazards recommended that “all countries should consider a combination of response measures: case and contact finding; containment or other measures that aim to delay the onset of patient surges where feasible; and measures such as public awareness, promotion of personal protective hygiene, preparation of health systems for a surge of severely ill patients, stronger infection prevention and control in health facilities, nursing homes, and long-term care facilities, and postponement or cancellation of large-scale public gatherings.” ^4^ Preparedness to support these activities, and the strategies used, have varied. Whilst recognising that different contexts require different approaches ^5^, we aimed to develop criteria to identify countries that have limited the impact of COVID-19 as “success stories” with valuable lessons to share to other countries.

## Methods

We assembled a group with expertise in public health policy, infectious diseases, outbreak response, and data analytics to develop a process to identify countries with emerging success in their response to COVID-19.

### Countries from which others can potentially learn

To be considered as an emerging success in our analysis, we only included countries meeting three key criteria. Firstly, to ensure that results in indicators were unlikely to be due to chance, and generalisable, we only included countries with populations over 5 million people. Secondly, we wanted to include countries with sufficient cases to demonstrate success, whilst recognizing that in some countries, low prevalence may be the result of effective containment, so we only included countries which had had at least 100 confirmed cases 21 days before our analysis. Thirdly, although data on COVID-19 cases and deaths are widely available, data on testing are less so and these data are essential to our analysis across the detect, contain, and treat phases, so we only included countries publishing testing data.

We used these three criteria as a first filter to identify countries with emerging success from all countries worldwide. Data were extracted on 18 ^th^ May 2020, from those curated by *Our World In Data* ( https://ourworldindata.org/coronavirus), based on daily published figures of cases and deaths from the European Centre for Disease Control. Population adjustments for per capita comparisons were made based on population data from the United Nations Population Division ^6^. The database on testing was curated by Our World in Data based on information from public sources, including official websites, in press releases and by social media accounts of national authorities—usually governments, ministries of health, or centres for disease control ^7^.

### Countries with indicators of success in response to COVID-19

We then considered indicators which could reflect successful interventions to COVID-19. The Global Health Security Index (GHSI) is a tool to assess countries’ health security, considering broad risks as well as geopolitical considerations, the health system, and whether the capacity to contain outbreaks has been tested ^8^. It describes six categories: prevention, detection, rapid reporting, health system, compliance with international norms, and risk environment. Because transmission of COVID-19 is ongoing, it is too soon to determine if any country will ultimately succeed at prevention, so we excluded the prevention phase from our analysis and focused on three categories: detect, contain and treat. We cross-referenced these three categories to key actions from the WHO’s “Strategic preparedness and response plan for the novel coronavirus” and then, based on the key actions, we selected indicators specific to COVID-19 that could be used to assess success in a country’s response ( Table 1) ^9^.

**Table 1.** Indicators for evaluating country performance cross referenced against the Global Health Security Index, WHO response pillars and the phases of response.

GHSI	Response phase ^10^	Key actions	WHO Pillars	Indicators	Exemplars
**Prevent**	Prepare		1,5		
**Detect**	Contain	Detection of cases	1,4,5 1,3,4	Total COVID-19 tests per 1,000 people Number of COVID-19 tests per confirmed case Total COVID-19 tests vs. Confirmed deaths per million people	**Detect**
**Respond**	Contain	Isolation of suspected cases, contact tracing	1,6,7	Total confirmed COVID-19 cases per million people Total confirmed COVID-19 cases per million vs. Doubling time of total confirmed cases (7-day period) Total confirmed COVID-19 deaths per million people Total confirmed COVID-19 deaths per million vs. Doubling time of confirmed deaths (7-day period),	**Contain**
**Healthcare**	Mitigate	Case management Health care capacity	1,7,8	Case-fatality rate of the ongoing COVID-19 pandemic Total confirmed COVID-19 deaths vs. cases	**Treat**

WHO Pillars Pillar 1- Country level coordination, planning, monitoring Pillar 2 – Risk communication and community engagement Pillar 3 - Surveillance, Rapid Response Teams, And Case Investigation Pillar 4 - Points of Entry Pillar 5 – National laboratories Pillar 6 - Infection Prevention and Control Pillar 7 - Case Management Pillar 8 - Operational Support and Logistics GHSI=Global Health Security Index; WHO=World Health Organization

### Principles for comparison

In doing this, we followed some general principles to ensure fair comparisons could be made between countries. Firstly, we used per capita indicators to account for the country’s population. Secondly, we primarily used absolute dates (calendar dates) instead of relative dates (for example the number of days since a certain number of deaths), reflecting the fact that all countries were aware of events worldwide at the same time.

***Detect***. Testing strategies may differ during an outbreak. For example, when there are fewer cases and the aim is to contain the outbreak by contact tracing, a high number of tests per case may increase the chance of successful containment. If the outbreak has progressed to widespread community transmission and it is not feasible to trace all contacts of positive cases, then high levels of testing may not be as critical for community control. In this situation, in many places, population-wide restrictions are in place to reduce spread across the population rather than specifically targeting contacts of confirmed cases. There are many challenges with counting cases, not least because this will depend on the availability of testing and the ability to detect those with no or mild symptoms ^9^. Counting and detecting deaths is likely to be a more accurate measure of the spread of the infection than cases. Based on these considerations, we defined key indicators for the “Detect” phase as **i) the number of tests per capita against time**, **ii) the number of tests per confirmed case**, and **iii) the number of tests per confirmed death with COVID-19 per capita**.

***Contain***. Although identifying people with COVID-19 depends on a country’s testing strategy and testing capacity, relative to its population, we felt that review of case data was still important. Case doubling time may be less affected by testing capacity than case per capita, as long as testing capacity has not changed substantially or is at its maximum. However, as testing capacity has indeed changed over time, which may be reflected in an increase in cases, we also included deaths in our indicators for the “Contain” phase, since these are likely to have been tested and recorded more completely than cases. We included people with confirmed COVID-19 infection (cases) as an outcome in terms of **iv) confirmed cases per capita against time** and **v)confirmed cases per capita compared to confirmed case doubling time (7-day period)**, and people with confirmed death from COVID-19 in terms of **vi) confirmed COVID-19 deaths per capita** and **vii) confirmed COVID-19 deaths per capita compared to confirmed death doubling time (7-day period)**.

***Treat***. There are many reporting biases when calculating the case fatality rate (CFR) ^11, 12^. For example, the number of cases (denominator) will depend on the number of tests being done in total, and who is being tested. Countries testing many people, or which include those who are asymptomatic or mildly ill (an infection fatality rate), will appear to have a lower CFR than countries testing only those who seek health care or are hospitalised, for example. There is also a delay between when people are infected and when they die. In countries going towards the epidemic peak, CFR will be underestimated because many people who will eventually die are still in the community or are hospitalised with severe infection ^13^. There are also true demographic differences; countries with older populations or a high proportion of comorbidities have more people vulnerable to severe disease, and thus higher CFRs may be due to this, rather than inadequate treatment ^14, 15^. While recognising these issues, we selected indicators to assess efforts to limit the number of deaths relative to the number of cases detected and how these changed over time; **viii) case fatality rate over time** and **ix) confirmed COVID-19 deaths compared to cases**.

### Countries representing diverse regions and key intrinsic factors

We examined the countries that met our inclusion criteria against each of the nine indicators at specific time points (i.e., fixed calendar dates) to reflect, for example, countries with high testing capacity early on in the outbreak (January and February 2020), and countries moving through the phases of the outbreak. From this shortlist, we selected three countries with emerging success stories, also reflecting diverse geographic regions and varied intrinsic factors such as land and sea borders and differing political structures. This final decision for selection was to support learning from these countries relevant to as many other countries as possible.

## Results

### Countries from which others can potentially learn

**Figure 1.**
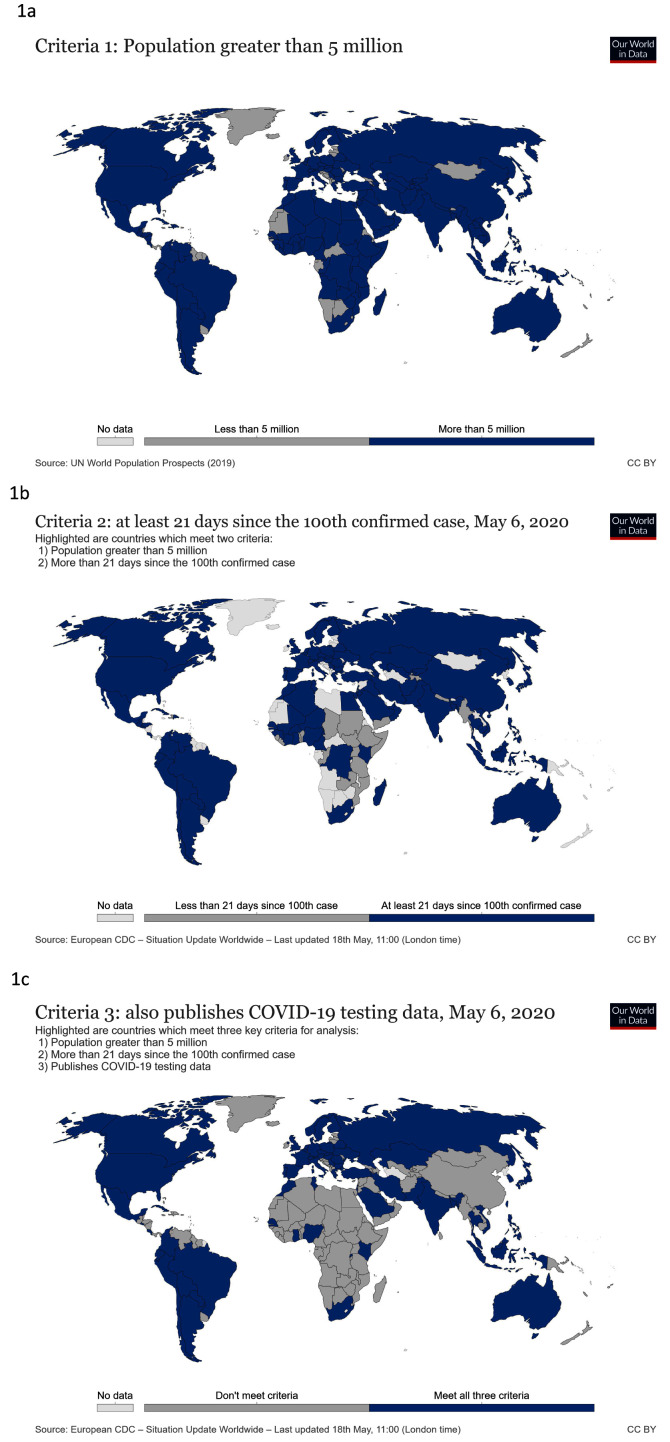
Countries assessed in this study. These world maps show countries with **a**) populations over 5 million people, **b**) at least 100 confirmed people with COVID-19 21 days prior to assessment and **c**) published testing data. Updated graphs can be found here, here and here.

On May 18 ^th^ 2020, 123 countries met the population size criterion ( Figure 1a), 98 met the total number of cases criterion ( Figure 1b) and 66 reported testing data ( Figures 1c). Although all African countries have reported cases of COVID-19, few have yet reached 21 days since their 100th confirmed case and even fewer are reporting testing data so the continent is under-represented in this analysis.

### Identifying countries with indicators of success

**Figure 2.**
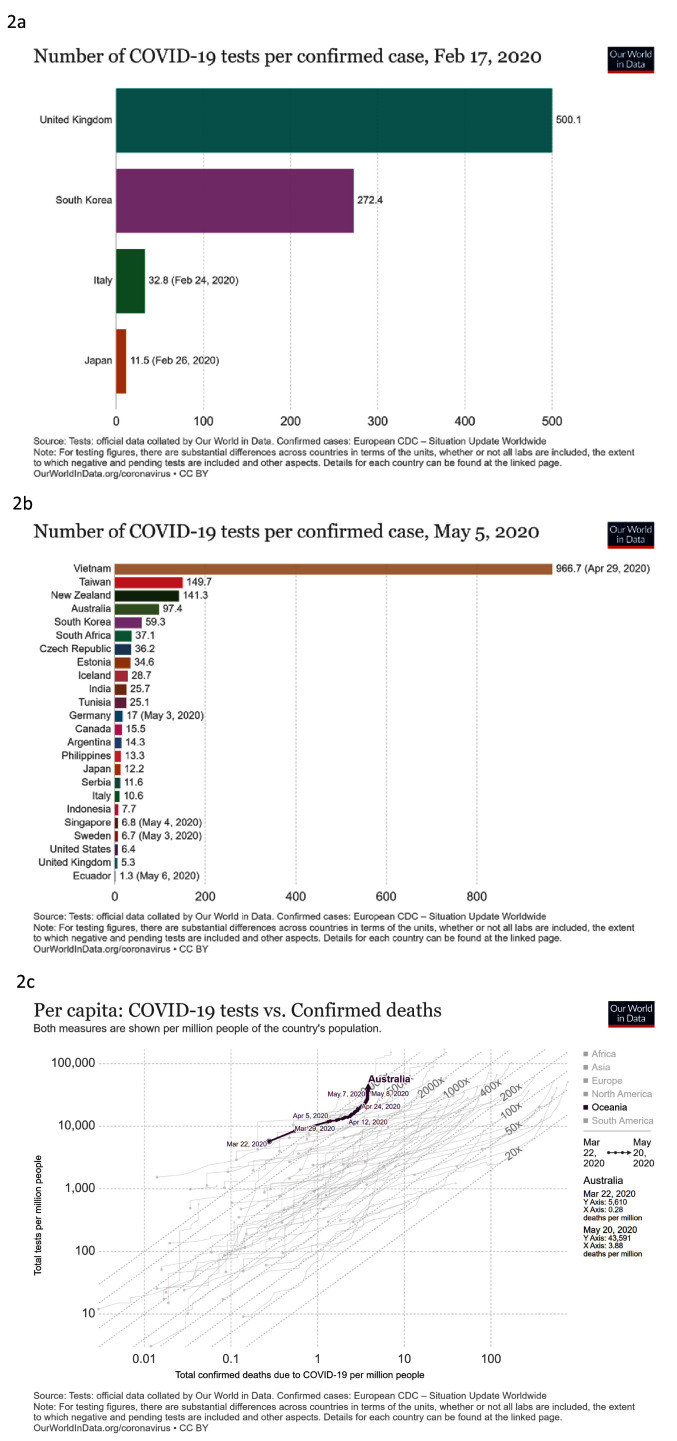
Detect. These graphs show **a**) total COVID-19 tests per 1,000 people **b**) number of COVID-19 tests per confirmed case of COVID-19 **c**) total COVID-19 tests compared to confirmed deaths with COVID-19 per million people. Updated graphs can be found here, here and here.

***Detect***. We looked at total COVID-19 tests per 1,000 people against time, number of COVID-19 tests per confirmed case and total COVID-19 tests against confirmed deaths per million people ( Figure 2). South Korea had high testing per confirmed case during January and February 2020, as did the United Kingdom. Capacity in South Korea continued to increase ( Figure 2a), but Germany had higher capacity than both South Korea and the UK in mid-March, as Europe was declared the COVID-19 epicentre. Germany’s testing continued to rapidly increase, from 7 to 29 tests per million people by the end of April. South Korea also had a high ratio of tests to confirmed cases early in the outbreak, decreasing as large clusters of infection developed during early March. In early May, Vietnam, was performing very strongly in terms of the number of tests per confirmed case ( Figure 2b). Australia also had high levels of testing, and when Australia recorded its first COVID-19 death, it had conducted 5,610 tests per million people, the highest among countries meeting our criteria ( Figure 2c).

**Figure 3.**
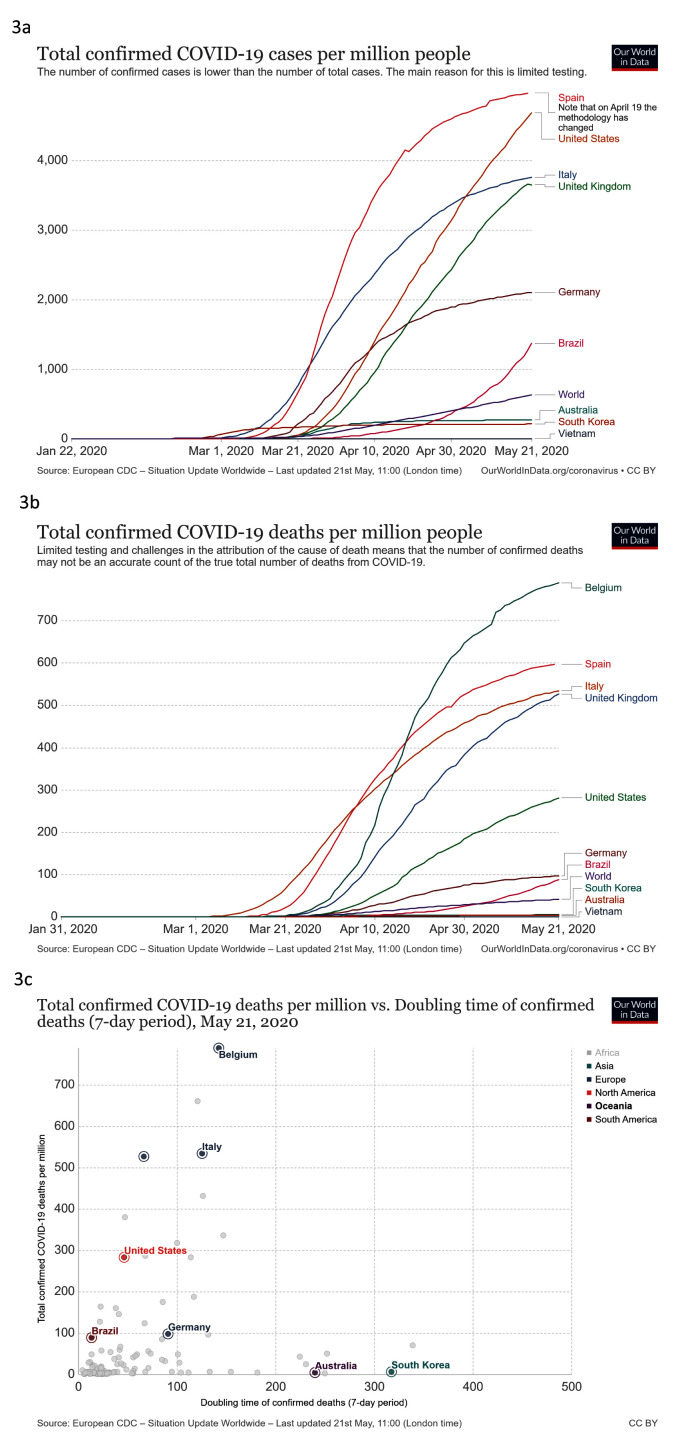
Contain. These graphs show **a**) total confirmed COVID-19 cases per million people **b**) total confirmed COVID-19 cases per million people compared to doubling time of total confirmed cases (7-day period) **c**) total confirmed COVID-19 deaths per million people against time **d**) total confirmed COVID-19 deaths per million compared to doubling time of confirmed deaths (7-day period). Updated graphs can be found here, here, here and here.

***Contain***. We looked at total confirmed COVID-19 cases per million people, confirmed COVID-19 cases per million against doubling time of total confirmed cases (7-day period), confirmed COVID-19 deaths per million people against time and confirmed COVID-19 deaths per million against doubling time of confirmed deaths (7-day period) ( Figure 3).

South Korea and Australia had very low cases of COVID-19 per capita (215 cases per million and 276 cases per million, respectively) compared to the rest of the world, but Vietnam was even lower, with only 3 cases per million ( Figure 3a). Cases have peaked at different times worldwide, and as the number of new cases begins to reduce across Europe and North America (April/May 2020), other countries are seeing rapid increases in confirmed cases, for example Brazil ( Figure 3a). For deaths, similar patterns emerge; Vietnam has not recorded any deaths with COVID-19 while South Korea and Australia have low numbers of deaths per capita compared to the rest of the world. This is in contrast to many European countries, with Belgium reporting the highest number of deaths per million people (786) ( Figure 3b). Some of the countries recording a very high number of deaths from COVID-19 have passed the peak cases and doubling times for deaths are increasing, for example in Italy. ( Figure 3c).

**Figure 4.**
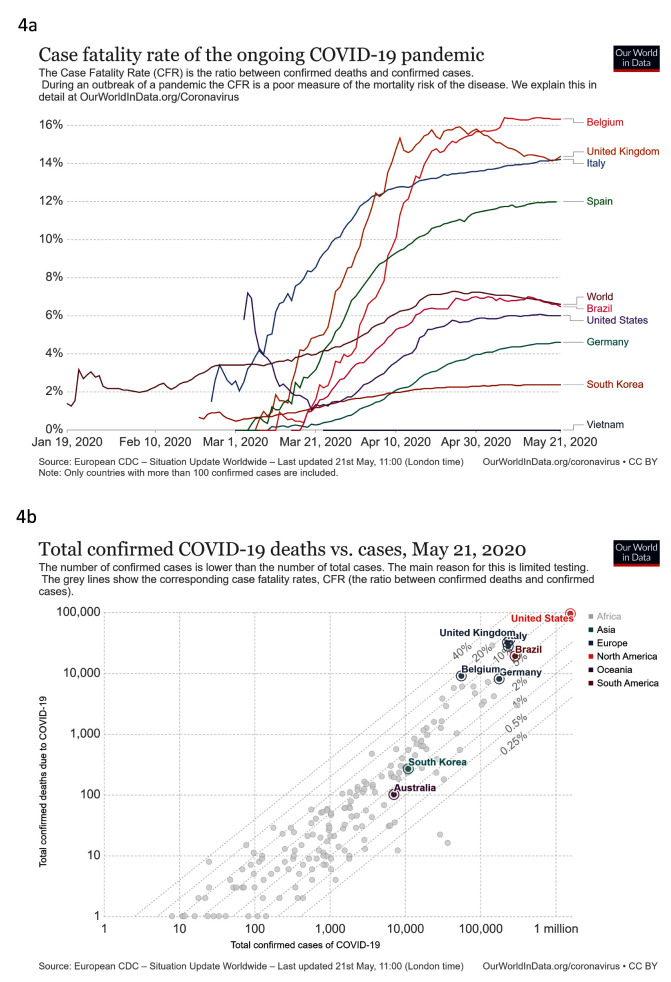
Treat. These graphs show **a**) case fatality rate against time **b**) Total confirmed COVID-19 deaths vs. cases. Updated graphs can be found here and here.

***Treat***. We looked at total confirmed COVID-19 deaths compared to cases, and case fatality rate (CFR) against time ( Figure 4). South Korea reported only 263 deaths from 11,065 cases, a CFR of 2.4%. Germany reported substantially more deaths (7,935) and cases (174,697), a CFR of 4.5%, below the global average of 6.7%. Neither country had an apparent rapid increase in CFR seen in several other countries. These increases may be caused by an inaccurate denominator for CFR due to low testing capacity in relation to increasing cases ( Figure 4a). South Korea and Australia have limited cases and deaths as shown in Figure 4b. While there are limitations to assessing the CFR in COVID-19 ^16^, the high levels of testing in South Korea and Germany will increase accuracy compared to settings with lower testing.

***Overall***. Several countries have indicators of success and as the pandemic continues to evolve, success will be found in other countries. Through the process we applied, we identified Vietnam, South Korea, and Germany as three countries with emerging success stories. Vietnam has had high levels of testing and effective containment ^17^. South Korea has also had high levels of testing and effective containment. Whilst Germany had a relatively large outbreak compared to South Korea and Vietnam, the number of cases and deaths per capita rose more slowly than other more similar European countries, and not to the same heights, suggesting success across detection, containment and treatment.

## Conclusions

We developed selection criteria to identify countries with indicators of success across current activities in relation to the COVID-19 outbreak. Several countries performed strongly in and across the three phases of detect, contain and treat. We identified Vietnam, South Korea and Germany as countries with indicators of success, and differing experiences, from whom lessons could be drawn. In these countries, high levels of testing early in the outbreak and continued increases in capacity with rising cases, appear to have supported containment. Both Vietnam and South Korea have had experience of other zoonotic coronaviruses ^18^, Severe acute respiratory syndrome coronavirus (SARS-CoV-1) and Middle East respiratory syndrome (MERS), respectively, which has likely supported their response to COVID-19 ^19^; both countries rapidly scaled up capacity for testing ^20^.

Although we sought to develop and apply objective indicators in our analysis, we recognize limitations to our process. We may have excluded countries from whom other particular lessons could be drawn. We did not include Australia in the final selection, for example, due to its particular geography. Other countries were excluded based on population size, for example Iceland and New Zealand. Iceland has very high testing per capita, and New Zealand quickly put in place widespread restrictions, and reported elimination of COVID-19 in May 2020 ^21^. Many African countries have not been included due to insufficient cases and limited availability of testing data at the time of analysis, who have used low-cost approaches ^22^. Other countries, such as Sweden, have aimed to increase population-level immunity through community-wide infection to avoid a second wave of infection, and the success of this will only be known with time.

We assessed countries as single units, but recognize that there can be variations within a country; countries with large populations may not be comparable to smaller countries where the outbreak does not vary by subnational region. Within the United States, for example, each of the 50 states has different systems in place for testing and reporting. We also could not assess the details of testing strategies. In countries with widespread transmission, targeting testing at key groups such as health care workers or high-risk locations such as hospitals and care homes may be appropriate, but lead to lower apparent testing and/or higher apparent CFR. For countries coming out of widespread restrictions and returning to strategies of intensive testing and contact tracing, there are insufficient data as yet to assess success in avoidance of a “second wave” of infection.

It is also true that countries have very different challenges. Many factors unrelated to interventions affect the spread and impact of COVID-19. For example, there is high transmission in urban centres, where the outbreak will be harder to contain. Population demographics will also influence the case fatality rate (CFR) for COVID-19 which varies by sex, age, and the presence of comorbidities ^23, 24^. As well as true variation due to these factors, the variation in CFR between countries is highly subject to testing and reporting ^11^. Belgium, for example, has a comprehensive approach in its counting of deaths with COVID-19 (for example including those in care homes from the outset, where high proportions of deaths have occurred in many contexts) and, of the countries meeting our selection criteria, Belgium reported the highest number of deaths per million (692). In the longer-term, data on excess mortality (exceedance in number of actual deaths compared to expected deaths over a defined time period in a country) will provide comparative data to better assess a country's success in terms of mitigating the impact of the COVID-19 outbreak ^25^.

It is important to look for countries with emerging success stories now, despite the challenges in doing so. SARS-CoV-2 continues to spread worldwide and policy makers are faced with difficult decisions daily. Here we describe a systematic approach to identifying success, from the range of current metrics available and with consideration of their limitations. We are facing a global challenge. In many countries, it may be only just beginning. Looking for lessons, and sharing them, is critical.

## Data availability

### Source data

All data are publicly available and shared through Our World In Data and Exemplars in Global Health websites. A summarized version of the results of this work is shared on these websites https://ourworldindata.org/coronavirus and https:www.//exemplars.health/emerging-topics/epidemic-preparedness-and-response/covid-19/finding-covid-19-success-stories.

The underlying data and scripts used to produce all datasets by Our World in Data are made publicly available on GitHub under a Creative Commons Attribution 4.0 International (CC BY 4.0) license.
