# Supplementary material for: COVID-19: Identifying countries with indicators of success in responding to the outbreak
Source: Gates Open Res. 2021 Sep 29;4:62. Originally published 2020 Jun 18. [Version 2] doi: 10.12688/gatesopenres.13140.2 (PMC8515014; doi:10.12688/gatesopenres.13140.2)
Supplement: Peer review history

# Peer review: Reviewer response for version 2

**DOI:** 10.21956/gatesopenres.14650.r31223

**Published:** 2021-10-13

**Authors:** Flavio Codeco Coelho

**Affiliations:** 1 School of Applied Mathematics, Fundação Getulio Vargas, Rio de Janeiro, Brazil; 2 Institute of Global Health, University of Geneva, Geneva, Switzerland

I have no further comments.

Is the work clearly and accurately presented and does it cite the current literature?

Yes

If applicable, is the statistical analysis and its interpretation appropriate?

Not applicable

Are all the source data underlying the results available to ensure full reproducibility?

Yes

Is the study design appropriate and is the work technically sound?

Partly

Are the conclusions drawn adequately supported by the results?

Partly

Are sufficient details of methods and analysis provided to allow replication by others?

Yes

Reviewer Expertise:

Infectious disease epidemiology, Mathematical epidemiology

I confirm that I have read this submission and believe that I have an appropriate level of expertise to confirm that it is of an acceptable scientific standard.

# Peer review: Reviewer response for version 2

**DOI:** 10.21956/gatesopenres.14650.r31222

**Published:** 2021-09-29

**Authors:** Richard A. Kock

**Affiliations:** 1 Department of Pathobiology and Population Science, Royal Veterinary College, London, UK

No further comment.

Is the work clearly and accurately presented and does it cite the current literature?

Yes

If applicable, is the statistical analysis and its interpretation appropriate?

Not applicable

Are all the source data underlying the results available to ensure full reproducibility?

Yes

Is the study design appropriate and is the work technically sound?

Yes

Are the conclusions drawn adequately supported by the results?

Yes

Are sufficient details of methods and analysis provided to allow replication by others?

Yes

Reviewer Expertise:

Emerging Infectious Diseases of animal origin

I confirm that I have read this submission and believe that I have an appropriate level of expertise to confirm that it is of an acceptable scientific standard.

# Peer review: Reviewer response for version 1

**DOI:** 10.21956/gatesopenres.14328.r29571

**Published:** 2020-09-17

**Authors:** Flavio Codeco Coelho

**Affiliations:** 1 School of Applied Mathematics, Fundação Getulio Vargas, Rio de Janeiro, Brazil; 2 Institute of Global Health, University of Geneva, Geneva, Switzerland

The idea behind the study is very important: to rank countries by success in fighting their COVID-19 epidemics.

 However, the manuscript presents only raw data without any statistical modeling. It is fairly well established that factors such as Age and comorbidities are important in determining the development of symptoms and the risk of hospitalization and death. The authors acknowledge this in the discussion, but the comparison between countries would be fairer if it was based on incidence and mortalities stratified by Age class. Co-morbidities are harder to control for, without having individual case data.

Is the work clearly and accurately presented and does it cite the current literature?

Yes

If applicable, is the statistical analysis and its interpretation appropriate?

Not applicable

Are all the source data underlying the results available to ensure full reproducibility?

Yes

Is the study design appropriate and is the work technically sound?

Partly

Are the conclusions drawn adequately supported by the results?

Partly

Are sufficient details of methods and analysis provided to allow replication by others?

Yes

Reviewer Expertise:

Infectious disease epidemiology, Mathematical epidemiology

I confirm that I have read this submission and believe that I have an appropriate level of expertise to confirm that it is of an acceptable scientific standard, however I have significant reservations, as outlined above.

# Response

**Published:** 2021-09-27

**Authors:** Anna Seale

Many thanks for this Review. Whilst we recognize the importance of modelling to support better estimation of disease and/or infection fatality risks, for example, this would be a different undertaking to the work done here, which aimed to identify exemplar countries, rapidly, to support public health policy. However, we have updated the discussion to more clearly reflect this limitation in our work and reflect this important comment.

# Peer review: Reviewer response for version 1

**DOI:** 10.21956/gatesopenres.14328.r29306

**Published:** 2020-09-07

**Authors:** Richard A. Kock

**Affiliations:** 1 Department of Pathobiology and Population Science, Royal Veterinary College, London, UK

The article 'COVID-19:Identifying countries with indicators of success in responding to the outbreak' examines different responses to COVID-19 and relative success, looking to explain key factors that might be critical to that success. In order to handle the diversity in demograpics population minimums, and case minimums and data availability were used as a filter for anlaysis. Success was judged on detection rates, containment, and treatment of cases and outcomes. To enable comparison, data was standardised indicators per capita per million and over time. 3 countries were then selected as success stories showing diversity of region, demographic and governance. This is a relatively crude analysis but given the complexities in dealing with data comparabilities, well put together and it also recognises and states the weaknesses and need to re-examine the outcomes in the longer term. Nevertheless this analysis has shown considerable differences between states that might have been considered e.g. on GHSI to have similar strengths in the health systems and likely therefore to show similar outcomes. This was not the case and factors which determined success are not necessarily correlated with governance,  relative wealth and capacity. The importance of sustained and consistent policy was also demonstrated in the initial testing response of the UK (high) and subsequent dropping of this in favour of other actions which was associated with a relatively poor outcome in the end for this country especially with respect to the successful countries highlighted, which used testing as a mainstay from the outset.

 There are no errors that I can detect in the data presented but some sentences would benefit with a fresh look to ensure clear expression and explanation. There is one statement which I think needs revision and this is in the conclusion where reference is made of other zoonotic coronaviruses. As no animal source has been confirmed for SARS - 1 or SARS - 2 and given all known cases have been transmitted human to human or from fomites and there is confirmed evidence only for zooanthroponosis (reverse zoonosis), it would be more accurate to state the probable zoonotic origins of SARS viruses rather than a zoonosis per se. An alternative more accurate description would be an emerging human pathogen and disease with probable zoonotic origins (the WHO definition of zoonosis is infection directly transmissible from animals to humans with evidence of a permanent animal reservoir.) I recommend this article is indexed with minimal revision required.

 Correction to citation 18: R. A. Kock

Is the work clearly and accurately presented and does it cite the current literature?

Yes

If applicable, is the statistical analysis and its interpretation appropriate?

Not applicable

Are all the source data underlying the results available to ensure full reproducibility?

Yes

Is the study design appropriate and is the work technically sound?

Yes

Are the conclusions drawn adequately supported by the results?

Yes

Are sufficient details of methods and analysis provided to allow replication by others?

Yes

Reviewer Expertise:

Emerging Infectious Diseases of animal origin

I confirm that I have read this submission and believe that I have an appropriate level of expertise to confirm that it is of an acceptable scientific standard.

## References

[1] : The Global Health Security index and Joint External Evaluation score for health preparedness are not correlated with countries’ COVID-19 detection response time and mortality outcome. *Epidemiology and Infection*.2020; 10.1017/S0950268820002046 1-18 10.1017/S0950268820002046 PMC750617232892793

# Response

**Published:** 2021-09-27

**Authors:** Anna Seale

Many thanks for this Review. We have revised the manuscript according to the comments made.
